# Hepatic neuroendocrine neoplasm: imaging patterns

**DOI:** 10.1590/0100-3984.2019.0038

**Published:** 2020

**Authors:** Abdallah de Paula Houat, Augusto Castelli von Atzingen, Fernanda Garozzo Velloni, Rafael Andrade Santiago de Oliveira, Ulysses dos Santos Torres, Giuseppe D’Ippolito

**Affiliations:** 1 Escola Paulista de Medicina da Universidade Federal de São Paulo (EPM-Unifesp), São Paulo, SP, Brazil.; 2 Universidade do Vale do Sapucaí (Univás), Pouso Alegre, MG, Brazil.; 3 Diagnósticos da América S/A (Dasa), Barueri, SP, Brazil.; 4 Grupo Fleury, São Paulo, SP, Brazil.

**Keywords:** Carcinoma, neuroendocrine, Neuroendocrine tumors, Neoplasm metastasis, Liver, Carcinoma neuroendócrino, Tumores neuroendócrinos, Metástase neoplásica, Fígado

## Abstract

Neuroendocrine neoplasms (NENs) are a heterogeneous group of tumors with distinct morphological and biological manifestations, the liver being the main organ affected by its metastases. However, primary hepatic involvement is quite rare. Hepatic NENs can have a variety of radiological presentation forms and can therefore mimic other lesions, making their diagnosis challenging. Nonetheless, certain imaging aspects allow NENs to be included among the main differential diagnoses of hepatic lesions and can guide the search for an extrahepatic primary site when the probable diagnosis is metastases.

## Abstract (pt)

As neoplasias neuroendócrinas (NNEs) são um grupo heterogêneo de tumores com manifestações morfológicas e biológicas distintas, sendo o fígado o principal órgão acometido por suas metástases. Por outro lado, o acometimento primário deste órgão é muito raro. Podem-se observar as mais diversas formas de apresentação radiológica das NNEs hepáticas primária e secundária e, em razão dessa variedade e por mimetizar outras lesões, o seu diagnóstico pode se revelar um desafio. No entanto, alguns desses aspectos permitem incluir as NNEs entre os principais diagnósticos diferenciais de lesões hepáticas e, nos casos de metástases, orientar na busca de um eventual sítio primário extra-hepático.

## INTRODUCTION

Neuroendocrine neoplasms (NENs) are a heterogeneous group of tumors with diverse morphological and biological manifestations that may appear in any tissue, the gastrointestinal (GI) tract being its most common primary site, accounting for 66-74% of cases^(1)^. They can be associated with genetic factors (e.g., multiple endocrine neoplasia type I and Von Hippel-Lindau syndrome), although most NENs are sporadic tumors. In recent years, mainly due to advances in diagnostic methods, there has been an increase in the incidence of NENs in the United States, where it rose from 1.52 cases/100,000 population in 1973 to 7.41 cases/100,000 population in 2012^(2)^.

The diagnostic workup of an NEN includes hormone tests, imaging tests, and pathology studies. In patients with symptoms related to hormone production, biochemical tests should be requested to investigate the corresponding syndrome. For example, a 5-hydroxyindoleacetic acid test in a 24-h urine sample should be performed for carcinoid syndrome (serotonin hypersecretion). The diagnostic biomarker of choice for symptomatic and asymptomatic cases is chromogranin A because it is the most sensitive^(3)^. It is also an important biomarker for patient follow-up.

Collecting samples for anatomic pathology studies is of great importance for making the definitive diagnosis of an NEN. The access route will depend on the site affected. For example, endoscopic ultrasound is used for pancreatic neoplasms whereas percutaneous access is used when there is liver involvement. In the latter case, ultrasound-guided percutaneous access is preferred, although computed tomography (CT) guidance can be used in more difficult cases^(1)^.

The radiological methods used for investigating NENs include CT, magnetic resonance imaging (MRI), and nuclear medicine tests, especially positron emission tomography/CT (PET/CT) with ^18^F-fluorodeoxyglucose (^18^F-FDG) or ^68^gallium DOTATATE^(4)^. In cases of NENs, CT and MRI are used for locating and staging the disease, defining the extent of the primary lesion, and determining whether or not there are any metastases^(4,5)^. In most cases, regardless of their location, the lesions are hypervascular, hence the importance of the arterial phase in imaging protocols. However, depending on the type, size, and location of the tumor, the portal and late phases are also key for a more effective analysis. On MRI scans, in addition to a hypervascular enhancement pattern, lesions usually present a hyperintense signal on T2-weighted sequences and significantly restricted diffusion on diffusion-weighted imaging (DWI). The DWI technique, combined with the use of a hepatobiliary-specific contrast agent, has increased the sensitivity of MRI in detecting liver metastases, including those originating from NENs ^(4-6)^.

Images obtained with PET/CT are used for detecting and staging tumors, as well as for informing and monitoring the treatment of patients with an NEN. In addition to defining the location of the lesion, PET/CT scans provide us with physiological information represented by increased glucose metabolism or expression of somatostatin receptors, depending on the degree of tumor differentiation. The ^18^F-FDG radiotracer is mainly used to visualize the metabolic activity of poorly differentiated NENs, whereas ^68^gallium DOTATATE is used for well-differentiated tumors^(6)^.

Approximately 15% of patients with an NEN have metastases. Most (46-93%) of the metastases from an NEN affect the liver, whereas the pancreas and small intestine are the most common primary sites^(7)^. Primary hepatic NENs (PHNENs) are extremely rare, only approximately 150 cases having been reported to date (in the English-language literature). Diagnosing a hepatic NEN is challenging because its symptoms are unremarkable, indolent, and often nonspecific, as well as because its radiological findings are very similar to those of other liver lesions and their various forms of presentation^(1)^.

There are currently no definitive guidelines for the treatment of PHNENs and hepatic neuroendocrine metastases. The treatment plan is individualized, taking into account the site, stage, and degree of differentiation of the tumor, as well as the age, comorbidities, and symptoms of the patient, in a multidisciplinary approach in which surgery, chemotherapy, radiation therapy, transcatheter arterial chemoembolization, and the use of somatostatin analogues are all available options^(1,8,9)^.

Given the wide range of types and clinical characteristics of NENs, there is currently no consensus on how the affected patients should be monitored after treatment. Nevertheless, one could say that the follow-up of patients with liver metastases is individualized on the basis of their clinical status (including symptoms) and degree of tumor differentiation, making use of a combination of laboratory tests and tumor markers (including chromogranin A), as well as (anatomical and functional) imaging findings^(9)^.

In patients with a suspected PHNEN, one should try to confirm that there is no primary extrahepatic site. To that end, complementary tests, such as endoscopy, colonoscopy, and bronchoscopy, may be needed^(10)^.

The objective of this study was to illustrate the common and uncommon presentations of PHNENs and hepatic neuroendocrine metastases. We hope that the information provided will facilitate their diagnosis.

## PRIMARY HEPATIC NEUROENDOCRINE NEOPLASM (PHNEN)

Only 0.3% of all NENs are PHNENs. Although the histogenesis of a PHNEN is unknown, it is thought to originate from ectopic pancreatic or adrenal cells in the liver, neuroendocrine tissue in the intrahepatic biliary epithelium, or chronic inflammation in the biliary tract causing intestinal metaplasia^(10)^. This type of neoplasm can occur in patients of any age, although it is mainly reported in adults (40-50 years of age), with similar distribution between men and women^(11)^. There are no known risk factors for PHNEN, and the associated mortality rate is estimated to be 25%. In most cases (80%), it is not accompanied by metastases at the time of diagnosis^(1)^.

Because PHNENs are slow-growing tumors, with clinical manifestations that can be nonspecific or even absent, they are usually detected because of the appearance of symptoms caused by the mass effect in the liver and adjacent organs, such as jaundice, palpable mass, and abdominal distention, and pain. Approximately 5% of patients present with the classic carcinoid syndrome^(11)^. Establishing a diagnosis can be challenging because the radiological findings are quite similar to those of other liver lesions such as hepatocellular carcinoma, cholangiocarcinoma, metastatic liver disease, and hydatid cyst. One of the diagnostic criteria for a PHNEN is the absence of lesions at other sites commonly affected by this type of tumor, such as the small intestine, the pancreas, and the lungs^(12)^. Therefore, when an NEN is found in the liver, looking for an extrahepatic primary site is paramount^(10)^.

**Figure 1:**
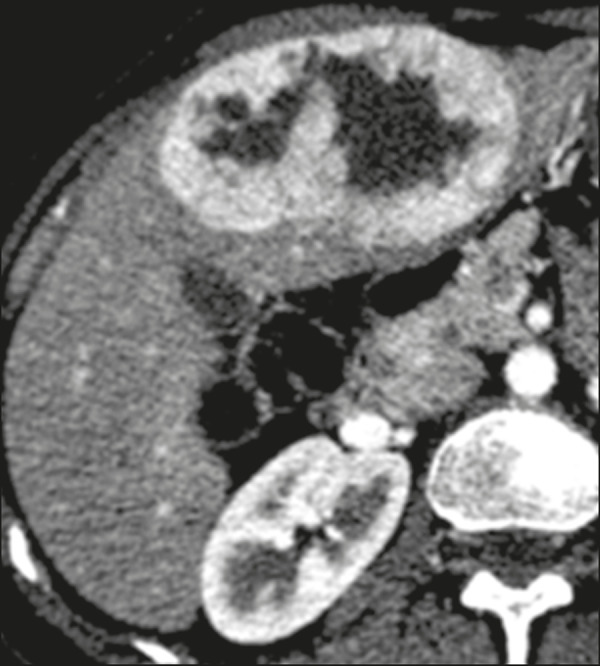
PHNEN. An arterial phase CT scan showing a large heterogeneous liver mass with a hypervascular enhancement pattern and a necrotic component.

**Figure 2:**
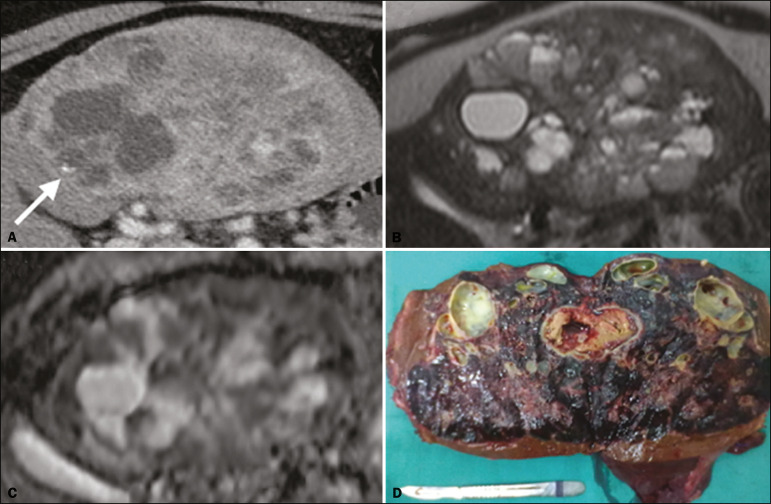
PHNEN. Arterial phase CT scan (**A**), T2-weighted MRI sequence (**B**), apparent diffusion coefficient map (**C**), and surgical specimen with multiple cysts (**D**). Note the large heterogeneous mass in the left lobe of the liver with a solid area showing a hypervascular enhancement pattern and a multicystic component characterized by hypoattenuating areas on the CT scan and areas of high signal intensity in the T2-weighted MRI sequence, some with a fluid-fluid level, and a punctate calcification focus (arrow in **A**). There is also markedly restricted diffusion in the solid area of the mass.

The radiological characteristics of PHNENs are not well known, probably because of the low number of cases reported. The lesions are usually single and heterogeneous, showing a hypervascular enhancement pattern that is more pronounced in the periphery and late enhancement in the center, with or without cystic areas (corresponding to necrosis) and a fluid-fluid level^(13,14)^, as depicted in Figures 1 and 2, respectively. In our experience, a fluid-fluid level has been a recurring feature, which we find curious.

## NEUROENDOCRINE HEPATIC METASTASES

Metastases from an NEN account for approximately 10% of all liver metastases. The liver is the organ most commonly affected by NEN metastases. Just over half of NENs of the GI tract show liver involvement at the time of diagnosis, and metastasis occurs, on average, seven years after the appearance of the primary lesion. The small intestine (terminal ileum) is the most common primary site^(7)^. The occurrence of metastasis depends mainly on the extent, degree of differentiation, and proliferative activity of the primary lesion. Liver metastases constitute the most important predictor of mortality in patients with an NEN. Among patients with an NEN, the five-year survival rate is significantly lower for those with liver metastases than for those without^(7,15)^-30-40% vs. 75-99%.

Although NENs of the GI tract have a slow progression, syndromic patients may present with symptoms related to hormone production and hypersecretion. In patients with nonfunctioning tumors, symptoms depend on tumor size and metastasis location, the most common clinical manifestation being nonspecific abdominal pain and the second most common being weight loss^(7)^.

**Figure 3:**
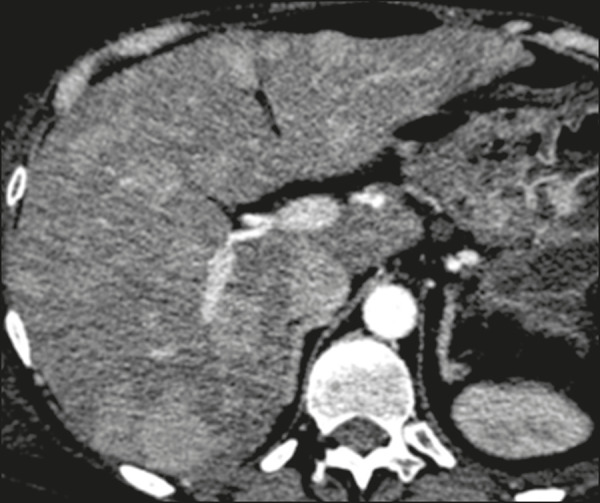
Arterial phase CT scan showing an NEN metastasis characterized by multiple liver nodules with a hypervascular enhancement pattern.

**Figure 4:**
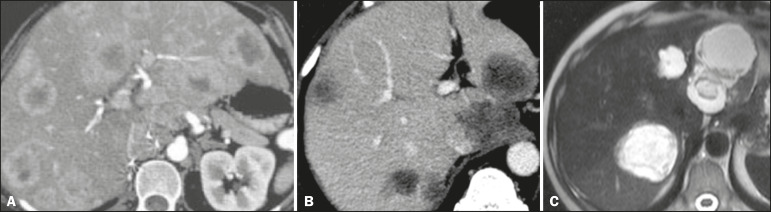
Possible differential diagnoses for metastases from an NEN. Liver metastasis from a renal cell carcinoma—arterial phase CT scan (**A**), from adenocarcinoma of the colon—arterial phase CT scan (**B**), and from sarcoma—T2-weighted MRI sequence (**C**).

**Figure 5:**
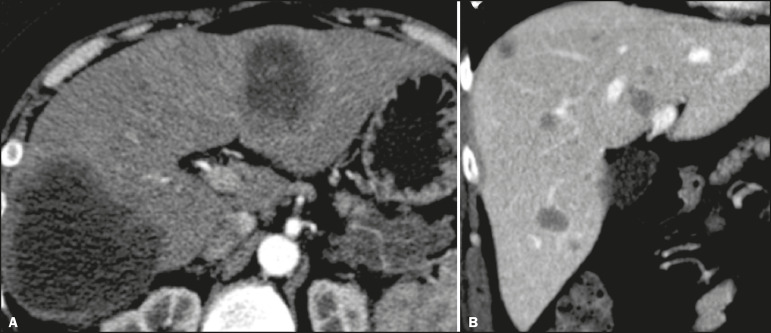
Arterial phase CT scan showing metastases from an NEN, characterized by masses (**A**) and multiple nodules (**B**) with a hypovascular enhancement pattern.

Metastatic lesions from NENs typically present as multiple hypervascular nodules^(15)^ (Figure 3). They are similar to metastases from thyroid carcinomas, melanomas, and renal cell carcinomas (Figure 4A). Metastases from an NEN can also present as hypovascular masses or nodules (Figure 5). Larger lesions can have a cystic appearance because of the liquefaction component resulting from tumor necrosis^(15)^ (Figure 5A). Other tumors that present the same pattern are metastases from the GI tract (Figure 4B), from the lungs, and from sarcomas.

**Figure 6:**
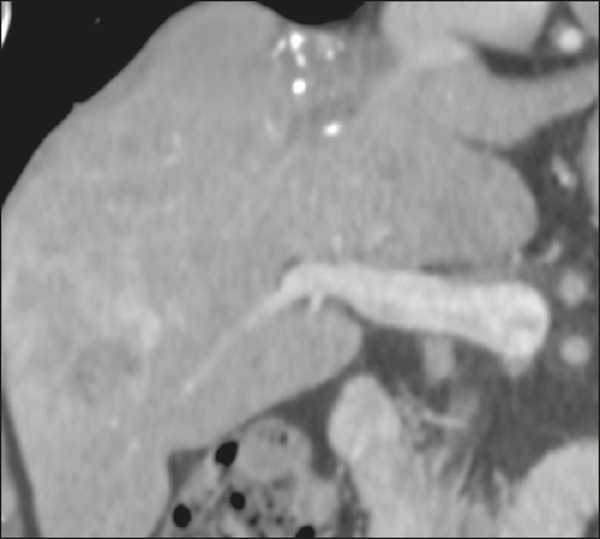
T2-weighted MRI sequence showing liver metastases with a cystic component exhibiting a fluid-fluid level.

**Figure 7:**
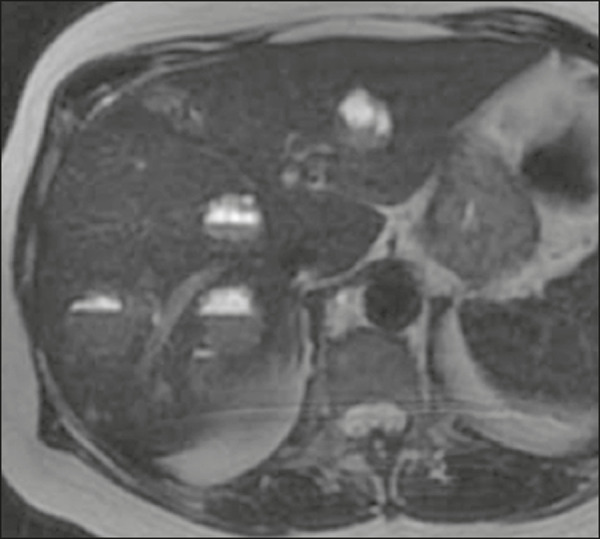
Portal phase CT scan showing a liver metastasis containing foci of calcification.

In a metastasis from an NEN, a calcification component (Figure 6) is uncommon, only a few cases having been reported to date^(16)^. In contrast, a hepatic lesion with a fluid-fluid level is highly suggestive of metastasis from an NEN (Figure 7), a finding that is probably caused by hemorrhage/high protein content related to the tumor hormone production^(17)^. However, a fluid-fluid level can also be seen in metastases from ovarian carcinoma and sarcoma (Figure 4C). Metastases from an NEN can also present perilesional fat deposition, which has been reported specifically in metastases from insulinoma. That could be related to the insulin effect; that is, inhibition of fatty acid oxidation and the consequent accumulation of triglycerides in hepatocytes^(18)^.

**Figure 8:**
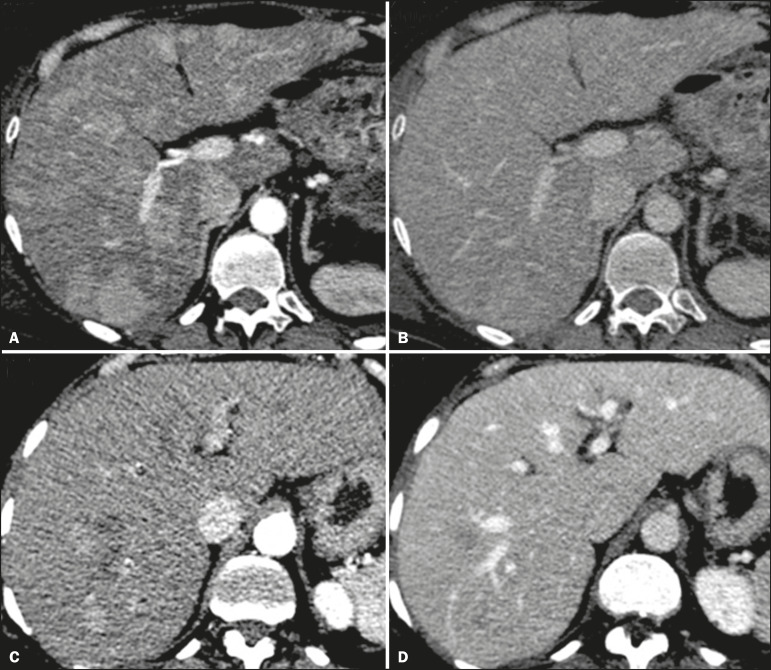
CT scans obtained in the arterial phase (**A,C**) and portal venous phase (**B,D**). Multiple liver nodules with a hypervascular enhancement pattern (**A,C**) and portal venous phase isointense attenuation in a case of a pancreatic NEN (**A,B**), and portal phase hypoattenuation in a case of an ileal NEN (**C,D**).

The enhancement pattern of a liver metastasis from an NEN can aid in the search for the primary site. For example, a hypervascular lesion with no washout in the portal venous phase can indicate that the primary site is in the pancreas (Figures 8A and 8B), whereas a hypervascular lesion with portal venous phase washout can indicate an enteric origin (Figures 8C and 8D)^(19)^.

**Figure 9:**
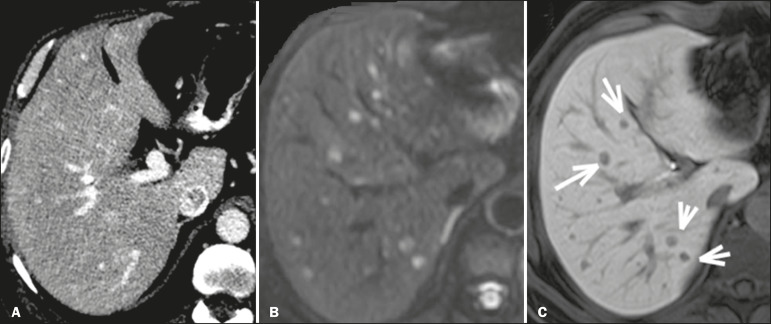
Arterial phase CT scan (**A**), DWI scan with a b value of 800 s/mm^2^ (**B**), and 20-min hepatobiliary phase MRI scan (**C**). The CT scan shows some sparse hypervascular nodules in the liver parenchyma. However, a close look at the images obtained with MRI using hepatobiliary-specific contrast, analyzing the DWI scan together with the hepatobiliary phase scan (arrows in **C**), reveals that there were multiple liver nodules.

In our experience, the use of DWI with a hepatobiliary-specific contrast agent, which has previously been shown to be a highly accurate method to detect liver metastasis in patients with colorectal cancer^(20)^, has proven to be more effective than is CT in detecting secondary liver lesions in the context of NENs^(6)^ (Figure 9).

## CONCLUSION

Because of their wide range of radiological presentations, PHNENs and neuroendocrine hepatic metastases can be mistaken for other liver lesions. There are, nevertheless, features that can hint at their origin, such as the presence of multiple hypervascular lesions coexisting with ileal or pancreatic masses (in cases of metastatic disease) or a single mass with cystic cavities and a fluid-fluid level (in cases of primary disease). The role of radiologists goes beyond diagnosis to include participation in the management, treatment, and follow-up of these neoplasms.
